# Factors associated with fatal cases of acute respiratory infection (ARI) among hospitalized patients in Guatemala

**DOI:** 10.1186/s12889-019-6824-z

**Published:** 2019-05-03

**Authors:** Sara Tomczyk, John P. McCracken, Carmen Lucia Contreras, Maria Renee Lopez, Chris Bernart, Juan Carlos Moir, Kenneth Escobar, Lisette Reyes, Wences Arvelo, Kim Lindblade, Leonard Peruski, Joe P. Bryan, Jennifer R. Verani

**Affiliations:** 1 Division of Global Health Protection, Centers for Disease Control and Prevention, Atlanta, USA; 2 Center for Health Studies, Universidad del Valle de Guatemala, Guatemala City, Guatemala; 3 Quetzaltenango Health Area, Ministry of Public Health and Social Welfare, Quetzaltenango, Guatemala; 4 Western Regional Hospital San Juan de Dios, Ministry of Public Health and Social Welfare, Quetzaltenango, Guatemala; 5 Santa Rosa Health Area, Ministry of Public Health and Social Welfare, Cuilapa, Guatemala; 6 Centers for Disease Control and Prevention, Nairobi, Kenya

**Keywords:** Acute respiratory infection, Death, Risk factors

## Abstract

**Background:**

Acute respiratory infection (ARI) is an important cause of mortality in children and adults. However, studies assessing risk factors for ARI-related deaths in low- and middle-income settings are limited. We describe ARI-related death and associated factors among children aged < 2 years and adults aged ≥18 years hospitalized with ARI in Guatemala.

**Methods:**

We used respiratory illness surveillance data in Guatemala from 2007 to 2013. ARI was defined as evidence of acute infection and ≥ 1 sign/symptom of respiratory disease in hospitalized patients. Clinical, sociodemographic, and follow-up data were gathered. Nasopharyngeal/oropharyngeal swabs were collected from patients with ARI and tested for 6 respiratory viruses; urine was collected only from adults with ARI and tested for pneumococcal antigen. Blood cultures and chest radiographs were performed at the physician’s discretion. Radiographs were interpreted per World Health Organization guidelines to classify endpoint pneumonia (i.e. suggestive of bacterial pneumonia). Multivariable logistic regression was used to compare characteristics of patients with fatal cases, including those who died in-hospital or were discharged in a moribund state, with those of patients with non-fatal cases.

**Results:**

Among 4109 ARI cases identified in hospitalized children < 2 years old, 174 (4%) were fatal. Median age at admission was 4 and 6 months for children with fatal and non-fatal cases, respectively. Factors associated with fatality included low weight-for-age, low family income, heart disease, and endpoint pneumonia; breastfeeding and respiratory syncytial virus (RSV) detection were negatively associated with fatality. Among 1517 ARI cases identified in hospitalized adults ≥18 years, 181 (12%) episodes were fatal. Median age at admission was 57 years for adults with fatal and non-fatal cases. Low body mass index, male sex, kidney disease, and endpoint pneumonia were significantly more common among patients with fatal versus non-fatal cases.

**Conclusions:**

Our findings highlight some of the factors that must be addressed in order to reduce ARI-related mortality, including promotion of good nutrition, breastfeeding, management and prevention of chronic comorbidities, and poverty reduction. Although no specific pathogen increased risk for death, endpoint pneumonia was significantly associated with fatality, suggesting that the pneumococcal conjugate vaccine could contribute to future reductions in ARI-related mortality.

## Background

Acute respiratory infection (ARI) is a leading cause of death in children and contributes to a substantial amount of mortality in adults worldwide. It is estimated that 11–22% of deaths among children aged < 5 years and 3% of deaths among adults aged 15–49 years globally are due to ARI [1, 2]. In Central America, ARI is the fourth leading cause of death among people of all ages [2]. Efforts to reduce ARI burden should include strategies to prevent infections from occurring and to prevent death among patients who become infected.

The integrated Global Action Plan for the Prevention and Control of Pneumonia and Diarrhea provides a framework of key evidence-based interventions and an integrated approach to ending preventable pneumonia and diarrhea deaths [3]. The plan highlights the need to use data to identify groups at greater risk for death and to develop targeted approaches. The World Health Organization (WHO) and the Child Health and Nutrition Research Initiative have also recognized the identification of key risk factors for the development of severe and fatal pneumonia as a research priority, particularly in low- and middle-income countries [4]. Such data can be used to guide policy decisions and the prioritization of prevention interventions. Although the burden of ARI-related mortality is disproportionately high in low- and middle-income settings, studies assessing risk factors associated with ARI-related deaths in these settings remain limited [5]. Many studies are restricted to specific settings or populations (e.g. specific respiratory pathogens such as influenza A H1N1 during the global pandemic or limited assessment of variables only in young children) rather than all ARI-related mortality among both children and adults [6–16].

To inform efforts to prevent deaths due to ARI, we assessed factors associated with ARI-related mortality among children aged < 2 years and adults aged ≥18 years hospitalized with ARI, using data from a multisite surveillance system in Guatemala over a six-year period.

## Methods

### Setting

The Vigilancia Integrada Comunitaria (ViCo) surveillance system is a facility-based integrated surveillance system for respiratory, diarrheal, febrile and neurologic illness in Guatemala. The surveillance system, described in more detail elsewhere [17, 18], was established in 2007 by Universidad del Valle de Guatemala (UVG) in collaboration with the Guatemala Ministry of Public Health and Social Welfare and Centers for Disease Control and Prevention (CDC). ViCo has been implemented at three sites in in Guatemala: Santa Rosa Hospital (176 bed capacity including four pediatric and eight adult intensive care unit beds [ICU]) located 50 km southeast of the capital Guatemala City, Quetzaltenango Hospital (425 bed capacity including 22 pediatric and six adult ICU beds) located 120 km northwest of the capital, and the Guatemalan Institute for Social Security, a hospital in the capital Guatemala city (179 bed capacity and 10 pediatric and ICU beds; only children enrolled at this site).

### Data collection

At each site, surveillance nurses reviewed registers and emergency department logs to identify patients admitted with an acute infectious disease. These patients were screened for study eligibility as ARI patients. ARI was defined as evidence of acute infection and ≥ 1 sign/symptom of respiratory disease. Evidence of acute infection included fever (≥38 °C), hypothermia (< 35 °C), abnormal white blood cell count (< 5 years of age: < 5500 or > 15,000; ≥5 years of age: < 3000 or > 11,000), or abnormal white blood cell differential. Signs or symptoms of respiratory disease included tachypnea, cough, sputum production, pleuritic chest pain, hemoptysis, difficulty breathing, shortness of breath, and sore throat; in addition, for children < 2 years old, signs included chest indrawing, nasal flaring, noisy breathing, and difficulty eating, drinking, or breastfeeding.

Patients meeting the ARI case definition or their parents/guardians were interviewed by surveillance nurses to gather demographic and epidemiological information. A study nurse abstracted the clinical data from the medical chart. Study nurses collected nasopharyngeal (NP) and oropharyngeal (OP) swabs, urine samples (from patients aged ≥18 years; urine antigen testing has low specificity in children due to frequent nasopharyngeal colonization [19]), and, when possible, measured peripheral oxygen saturation using a pulse oximeter with the patient off oxygen. Blood cultures and chest radiographs (CXRs) were performed at the discretion of the treating physician. Digital images of CXRs were captured and reviewed by a panel of radiologists as per WHO guidelines for standardized interpretation of CXRs [20]; although the guidelines were developed for pediatric CXRs, the same criteria were applied for CXRs from patients of all ages. The images were classified as ‘end-point pneumonia’, suggestive of bacterial etiology, when lobar consolidation and/or effusion were observed; other classifications included ‘other consolidation/infiltrate’, ‘no consolidation/infiltrate/effusion’ or ‘uninterpretable’ [21]. Enrolled patients were followed during their hospital stay, and data on outcomes were captured by study nurses. Patients were classified as “discharged moribund” if the chart indicated that they were terminally ill at the time of discharge. Follow-up contact was attempted three to 6 weeks after discharge to assess post-discharge sequelae or death.

NP and OP swabs were stored in viral transport media at 4 °C and real-time reverse transcriptase polymerase chain reaction was used to test for adenovirus, parainfluenza virus 1/2/3, respiratory syncytial virus (RSV), influenza A and B, and human metapneumovirus at UVG following CDC protocols [22–24]. Urine specimens were tested for *Streptococcus pneumoniae* antigen using Binax NOW (Binax Inc., Scarborough, ME, USA) tests. Blood cultures were performed at on-site laboratories in each surveillance hospital using standard microbiologic methods as previously described [17].

### Ethics

The surveillance protocol received approval from the institutional review boards of UVG (Guatemala City, Guatemala) and CDC (Atlanta, GA, USA) and the National Health Ethics Committee of the Guatemala Ministry of Public Health and Social Welfare. Verbal consent was obtained from all patients prior to eligibility screening; written informed consent was obtained from all eligible patients willing to participate. Parents or guardians provided consent for patients < 18 years.

### Data management and analysis

All data were entered into hand-held personal digital devices with pre-programmed quality checks and stored using Microsoft SQL Server 2008 (Redmond, VA, USA). ARI patients enrolled from September 2007 through December 2013 were included in the analysis. Certain variables were available only for children aged < 2 years (e.g. breastfeeding, prematurity) and others only for adults ≥18 years (e.g. history of patient smoking), so children aged 2 to 17 years were not included in the analysis; 8% (*n* = 31) of all ARI patients with fatal cases were in this age group. Malnutrition in children aged < 2 years was defined as weight-for-age Z score < − 2 using WHO growth curves [25]; low body mass index (BMI) in adults was defined as < 18.5 kg/m^2^ [26]. Family monthly income was measured according to the Guatemala currency Quetzal (Q). Patients who self-discharged against medical advice, were transferred, or who had missing discharge status were excluded from the analysis. Cases in which the ARI patient died in-hospital or was discharged home in a moribund condition were classified as fatal cases. Moribund patients were included based on an analysis of follow-up data 30 days post-discharge showing that 86% (19/22) of ARI patients aged < 2 years discharged in a moribund condition died post-discharge as compared to 0.6% (11/1876) of those discharged in a non-moribund condition (unpublished data). Likewise, 94% (29/31) of ARI patients aged ≥18 years discharged in moribund condition died post-discharge as compared to 3% (26/906) of those discharged in a non-moribund condition.

Characteristics of ARI patients with fatal cases were compared to those of patients with non-fatal cases, using a chi-squared test for categorical variables and a Wilcoxon-Mann-Whitney test for non-normal continuous variables. Pathogen specific case fatality ratios with 95% confidence intervals (CIs) were calculated. Multivariable logistic regression was used to assess risk factors for fatality among ARI patients. Models were developed using manual selection based on univariate effects, statistical significance (*p* < 0.1), and biologic plausibility (e.g. age was kept in the model a priori). Variable correlation was assessed. Interaction between age and pathogens was also tested. Models were evaluated using fit statistics (i.e. Akaike’s information criterion and Schwarz criterion), the Hosmer and Lemeshow Goodness of Fit Test, the C statistic, and an assessment of outliers. SAS version 9.3 (SAS Institute, Inc., Cary, NC, USA) was used for all analyses.

## Results

### Children aged < 2 years

**Fig. 1:**
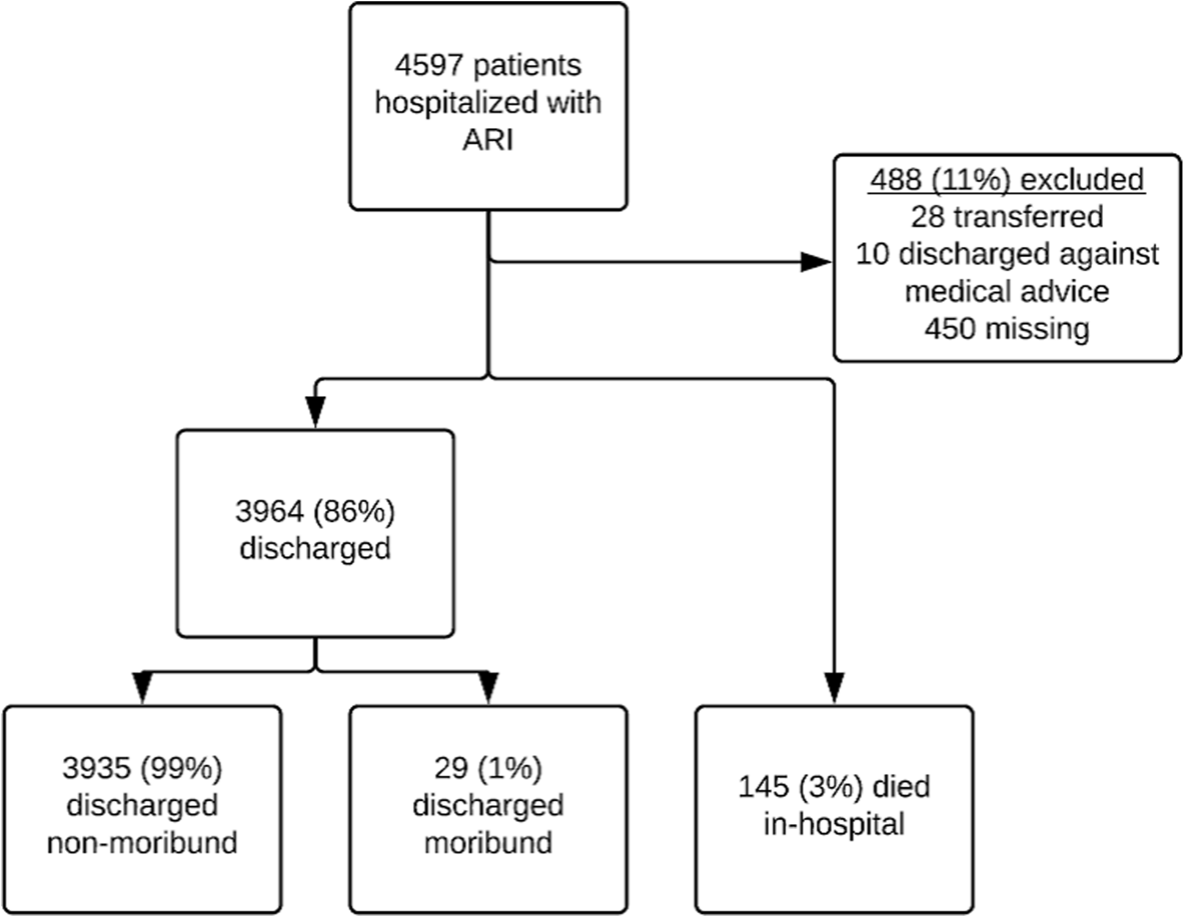
Children aged < 2 years hospitalized with acute respiratory infection (ARI) according to discharge status and related deaths in Guatemala from September 2007 through December 2013 (*N* = 4597)

From September 2007 to December 2013, 4597 children aged < 2 years old were hospitalized with ARI. After excluding 488 (11%) patients who transferred, discharged against medical advice, or had missing status at discharge, a total of 4109 ARI cases were available for analysis, of which 174 (4%) were fatal (including 145 in-hospital deaths and 29 cases in patients discharged moribund) (Fig. 1). Median age of patients with fatal and non-fatal ARI cases was four and 6 months, respectively; over half in both groups were male (Table 1). More than 96% of patients with both fatal and non-fatal cases had a NP/OP swab collected; at least one virus was detected in 53% of patients with fatal cases and 69% of patients with non-fatal cases (*p* < 0.0001). Blood culture was performed for 33 and 20% of patients with fatal and non-fatal cases, respectively (p = < 0.0001); very few respiratory pathogens were detected by blood culture among children (Table 1). Chest radiography was performed on 56% of enrolled ARI patients; 49/104 (47%) patients with fatal cases and 562/2197 (26%) patients with non-fatal cases had endpoint pneumonia.

**Table 1:** Patient characteristics and pathogens associated with fatality in children aged < 2 years hospitalized with acute respiratory infection (ARI) in Guatemala from September 2007 through December 2013 (*N* = 4109)

Variable	Fatal (%) (*n* = 174)	Non-fatal (%) (*n* = 3935)	Crude OR (95% CI)	Adjusted OR (95% CI)
Age at admission in months, median (IQR)	4 (2–9)	6 (2–11)	1.0 (0.9–1.0)	–
Male sex	105/174 (60)	2280/3935 (58)	1.1 (0.8–1.5)	–
Family monthly income <Q1000	123/174 (71)	2015/3935 (51)	2.2 (1.7–3.2)	2.2 (1.4–3.6)
Amerindian Indigenous	61/174 (35)	1227/3935 (31)	1.2 (0.9–1.6)	–
Hospital Site				
Santa Rosa	91/174 (52)	1561/3935 (40)	Reference	
Quetzaltenango	72/174 (41)	1484/3935 (38)	0.8 (0.6–1.1)	–
Guatemala City	11/174 (6)	890/3935 (23)	0.2 (0.1–0.4)	–
High crowding index (> 3 persons/room)	88/174 (51)	1900/3935 (48)	1.1 (0.8–1.5)	–
Parent completed primary school	72/173 (42)	2114/3919 (54)	0.6 (0.4–0.8)	–
A person in the household smokes	31/171 (18)	797/3898 (20)	0.9 (0.6–1.3)	–
Breastfed in first 2 years of life	72/172 (42)	2101/3924 (54)	0.6 (0.5–0.9)	0.6 (0.4–0.9)
Sought care prior to hospitalization	121/173 (70)	2570/3879 (66)	1.2 (0.9–1.7)	–
Received ≥1 dose of DPt/Hib, Hep B vaccine^a^	73/137 (53)	2231/3312 (67)	0.6 (0.4–0.8)	–
Received influenza vaccine in past 6 months, n (%)	2/127 (2)	76/2837 (3)	0.6 (0.1–1.9)	–
Low weight-for-age Z score (<−2 SD), n (%)	149/174 (86)	1563/3935 (40)	9.0 (6.0–14.2)	7.0 (4.1–12.8)
Medical history				
Any chronic disease	26/170 (15)	219/3857 (6)	3.0 (1.9–4.6)	–
Heart disease	19/165 (12)	108/3859 (3)	4.5 (2.6–7.4)	3.0 (1.2–6.8)
Asthma	2/169 (1)	57/3879 (1)	0.8 (0.1–2.6)	–
Chronic pulmonary disease	2/166 (1)	24/3855 (1)	1.9 (0.3–6.6)	–
Diabetes	0/167 (0)	3/3870 (0.1)	–	–
Cancer	0/166 (0)	1/3861 (0.03)	–	–
Liver disease	0/166 (0)	5/3852 (0.1)	–	–
Kidney disease	0/166 (0)	7/3856 (0.2)	–	–
HIV	0/159 (0)	3/3809 (0.1)	–	–
Prematurity	48/168 (29)	1049/3872 (27)	1.1 (0.8–1.5)	–
Endpoint pneumonia	49/104 (47)	562/2197 (26)	2.6 (1.7–3.9)	2.5 (1.6–3.8)
Pathogens detected by NP/OP swab				
NP/OP swab tested	169/169 (100)	3780/3781 (99)		
Any viral detection^b^	90/169 (53)	2609/3780 (69)	0.5 (0.4–0.7)	–
Respiratory syncytial virus^b^	50/169 (30)	1632/3780 (43)	0.6 (0.4–0.8)	0.5 (0.3–0.8)
Adenovirus^b^	23/169 (14)	399/3780 (11)	1.3 (0.8–2.0)	–
Parainfluenza 1/2/3^b^	14/169 (8)	445/3780 (12)	1.9 (1.0–3.9)	–
Influenza A^b^	14/169 (8)	179/3780 (5)	1.8 (1.0–3.1)	–
Human metapneumovirus^b^	9/169 (5)	357/3780 (9)	0.5 (0.3–1.0)	–
Influenza B^b^	1/169 (0.6)	40/3780 (1)	0.6 (0.03–2.6)	–
Pathogens detected by blood culture				
Blood culture performed	57/174 (33)	805/3935 (20)		
Evidence of contamination^b^	4/57 (7)	84/805 (10)	0.6 (0.2–1.6)	–
Any growth^b^	13/57 (23)	173/805 (21)	1.1 (0.5–2.0)	–
*Streptococcus pneumoniae*^b^	0/57 (0)	0/805 (0)	–	–
*Pseudomonas aeruginosa*^b^	0/57 (0)	3/805 (0.4)	–	–
*Staphylococcus aureus*^b^	0 (0)	21/805 (3)	–	–

Abbreviations: *IQR* interquartile range

^a^0/29 patients aged < 2 months with fatal cases and 39/674 patients aged < 2 months with non-fatal cases were vaccinated, although they were not yet age-eligible to receive DPt/Hib, Hep B vaccine

^b^Among those tested

On multivariable analysis, the factor most strongly associated with death was low weight-for-age Z score (adjusted odds ratio [aOR]: 7.0; 95% confidence intervals [CI]: 4.1–12.8), with 86% of patients with fatal cases having a low weight-for-age Z score compared to 40% of patients with non-fatal cases. Other factors significantly associated with death included family monthly income <Q1000 (aOR: 2.2; 95% CI: 1.4–3.6), a history of heart disease (aOR: 3.0; 95% CI: 1.2–6.8), and endpoint pneumonia (aOR: 2.5; 95% CI: 1.6–3.8). Breastfeeding in the first 2 years of life (aOR: 0.6; 95% CI: 0.4–0.9) and RSV detection (aOR: 0.5; 95% CI: 0.3–0.8) were significantly less common among patients with fatal cases compared with those with non-fatal cases (Table 1). Although neither agent was significantly associated with fatality, influenza A (7%) and adenovirus (5%) had the highest pathogen-specific case fatality ratios (Table 3).

### Adults aged ≥18 years

**Fig. 2:**
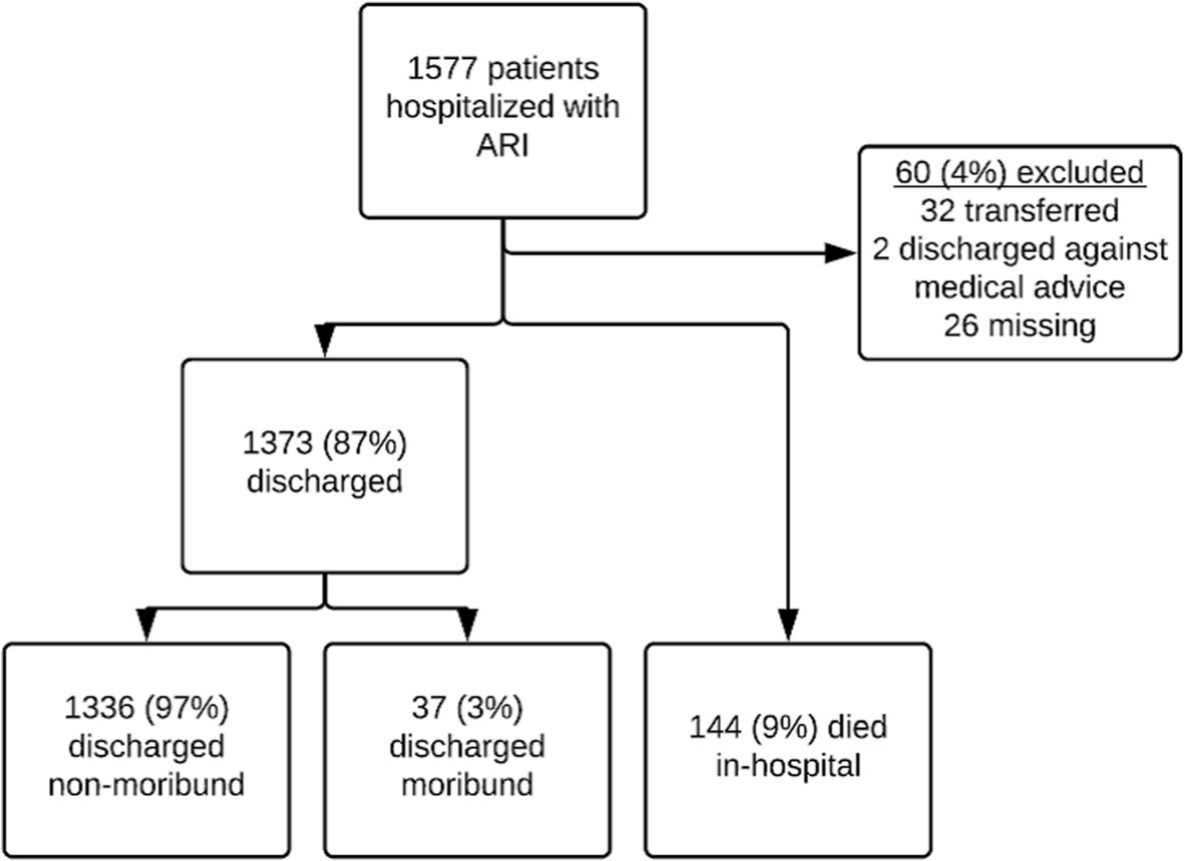
Adults aged ≥18 years hospitalized with acute respiratory infection (ARI) according to discharge status and related deaths in Guatemala from September 2007 through December 2013 (*N* = 1577)

During the study period, 1577 adults ≥18 years old were hospitalized with ARI. After excluding 60 (4%) patients who transferred, self-discharged against medical advice, or had missing status, a total of 1517 ARI cases were available for analysis, of which 181 (12%) were fatal (including 144 in-hospital deaths and 37 cases in patients discharged moribund) (Fig. 2). Median age of hospitalized ARI patients was 57 years; approximately half were male (Table 2). More than 97% of patients with both fatal and non-fatal cases had a NP/OP swab collected; at least one virus was detected in 29% of patients with fatal cases and in 27% of patients with non-fatal cases. Blood culture was performed for 26 and 21% of patients with fatal and non-fatal cases, respectively (*p* = 0.15) and pathogens detected are seen in Table 2. Chest radiography was performed on 63% of enrolled ARI patients; 73/117 (62%) of patients with fatal cases and 335/833 (40%) of patients with non-fatal cases had endpoint pneumonia.

**Table 2:** Patient characteristics and pathogens associated with fatality in adults aged ≥18 years hospitalized with acute respiratory infection (ARI) in Guatemala from September 2007 through December 2013 (*N* = 1517)

Variable	Fatal (*n* = 181) n (%)	Non-fatal (*n* = 1336) n (%)	Crude OR (95% CI)	Adjusted OR (95% CI)
Age at admission in years, median (IQR)	57 (39–74)	57 (39–71)	1.0 (1.0–1.0)	–
Male sex	93/181 (51)	570/1336 (43)	1.4 (1.0–1.9)	1.8 (1.2–2.7)
Family monthly income <Q1000	96/181 (53)	834/1336 (62)	0.7 (0.5–0.9)	–
Amerindian Indigenous	78/181 (43)	471/1336 (35)	1.4 (1.0–1.9)	–
Hospital Site				
Quetzaltenango	110/181 (61)	655/1336 (49)	1.6 (1.2–2.2)	–
Santa Rosa	71/181 (39)	681/1336 (51)	Reference	
High crowding index (> 3 persons/room)	44/181 (24)	322/1336 (24)	1.0 (0.7–1.4)	–
Patient completed primary school	60/180 (33)	396/1334 (30)	1.2 (0.8–1.6)	–
History of smoking	20/177 (11)	151/1331 (11)	1.2 (0.8–1.8)	–
Sought care prior to hospitalization	88/179 (49)	542/1335 (41)	1.4 (1.0–1.9)	–
Received influenza vaccine in past 6 months	6/156 (4)	33/1188 (3)	1.4 (0.5–3.2)	–
Low body mass index (< 18.5 kg/m^2^)	143/181 (79)	661/1336 (49)	3.8 (2.7–5.7)	3.4 (2.1–5.7)
Medical history				
Any chronic disease	99/180 (55)	666/1336 (50)	1.2 (0.9–1.7)	–
Hypertension	48/167 (29)	268/1303 (21)	1.6 (1.1–2.2)	–
Diabetes	31/174 (18)	182/1325 (14)	1.4 (0.9–2.0)	–
Kidney disease	26/174 (15)	79/1320 (6)	2.8 (1.7–4.4)	2.4 (1.3–4.3)
Heart disease	21/174 (12)	86/1320 (7)	2.0 (1.2–3.2)	–
Chronic pulmonary disease	13/174 (7)	105/1320 (8)	0.9 (0.5–1.6)	–
Asthma	9/176 (5)	137/1327 (10)	0.5 (0.2–0.9)	–
Liver disease	9/174 (5)	24/1321 (2)	2.9 (1.3–6.2)	–
HIV	8/166 (5)	22/1296 (2)	2.9 (1.2–6.4)	–
Cancer	3/175 (2)	20/1324 (2)	1.1 (0.3–3.4)	–
End-point pneumonia	73/117 (62)	335/833 (40)	2.5 (1.7–3.7)	1.9 (1.3–2.9)
Pathogens detected by NP/OP swab				
NP/OP swab tested	176/177 (99)	1292/1292 (100)		
Any viral detection^a^	51/176 (29)	343/1292 (27)	1.1 (0.8–1.6)	–
Influenza A^a^	18/176 (10)	116/1292 (9)	1.2 (0.7–1.9)	–
Adenovirus^a^	12/176 (7)	62/1292 (5)	1.5 (0.7–2.7)	–
Respiratory syncytial virus^a^	11/176 (6)	87/1292 (7)	0.9 (0.5–1.7)	–
Parainfluenza 1/2/3^a^	11/176 (6)	62/1292 (5)	1.3 (0.7–2.5)	–
Human metapneumovirus^a^	3/176 (2)	25/1292 (2)	0.9 (0.2–2.5)	–
Influenza B^a^	1/176 (0.6)	22/1292 (2)	0.3 (0.02–1.6)	–
Pathogens detected by blood culture				
Blood culture performed	47/181 (26)	284/1336 (21)		
Evidence of contamination^a^	1/47 (2)	8/284 (3)	0.8 (0.04–4.2)	–
Any growth^a^	13/47 (28)	38/284 (13)	2.5 (1.2–5.0)	–
*Streptococcus pneumoniae*^a^	3/47 (6)	11/284 (4)	1.7 (0.4–5.7)	–
*Staphylococcus aureus*^a^	2/47 (4)	3/284 (1)	4.2 (0.5–25.8)	–
*Pseudomonas aeruginosa*^a^	0/47 (0)	1/284 (0.4)	–	–
Pathogens detected by urine antigen testing				
Urine antigen testing conducted	134/163 (82)	1076/1188 (91)		
*Streptococcus pneumoniae*^a^	16/134 (12)	147/1076 (14)	0.9 (0.5–1.4)	–
Viral co-infection with *S. pneumoniae*^a, b^	5/178 (3)	50/1322 (4)	1.4 (0.6–4.0)	–

^a^Among those tested

^b^Results from blood culture and urine antigen testing combined

On multivariable analysis, the factor most strongly associated with death was low BMI (aOR: 3.4; 95% CI: 2.4–5.1), with 79% of patients with fatal cases having a low BMI, compared to 49% of patients with non-fatal cases. Other factors significantly associated with death included male sex (aOR: 1.6; 95% CI: 1.1–2.2), a history of kidney disease (aOR: 2.1; 95% CI: 1.2–3.4), and endpoint pneumonia (aOR: 1.8; 95% CI: 1.2–2.8) (Table 2). Although no pathogens were associated with fatality, adenovirus (16%), parainfluenza (15%), and influenza A (13%) had the highest case fatality ratios among viral pathogens. *S. pneumoniae* had a 10–21% case fatality ratio (see Table 3).

**Table 3:** Pathogen-specific case fatality ratios (CFR) among children aged < 2 years and adults aged ≥18 years hospitalized with acute respiratory infection (ARI) in Guatemala from September 2007 through December 2013 (*N* = 6385)

Pathogen	CFR in children < 2 years (%)	CFR in adults ≥18 (%)
Influenza A^a^	14/193 (7)	18/134 (13)
Adenovirus^a^	23/422 (5)	12/74 (16)
Parainfluenza 1/2/3^a^	14/459 (3)	11/73 (15)
Respiratory syncytial virus^a^	50/1682 (3)	11/98 (11)
Human metapneumovirus^a^	9/366 (2)	3/28 (11)
Influenza B^a^	1/41 (2)	1/23 (4)
*Staphylococcus aureus* ^b^	0/21 (0)	2/5 (40)
*Pseudomonas aeruginosa* ^b^	0/3 (0)	0/1 (0)
*Streptococcus pneumoniae* ^b^	–	3/14 (21)
*Streptococcus pneumoniae* ^c^		16/163 (10)
Viral co-infection with *S. pneumoniae*^d^	–	5/55 (9)

^a^Tested by NP/OP swab

^b^Tested by blood culture

^c^Tested by urine antigen testing

^d^Includes blood culture and urine antigen tested for adults ≥18 years

## Discussion

We found a substantial burden of mortality among hospitalized ARI patients, with 4% of patients < 2 years old and 12% of adult patients dying during hospitalization or discharged in a moribund state. Although the case fatality proportion among children was lower than that of adults, children hospitalized with ARI outnumbered adults hospitalized with ARI by almost three to one. We identified several factors associated with fatality among hospitalized ARI patients, including malnutrition, lack of breastfeeding, certain medical conditions, low socioeconomic status, and male sex, in these age groups. Although we did not find any specific pathogens to be positively associated with fatality, endpoint pneumonia, which is suggestive of bacterial pneumonia [20], was more common among patients with fatal ARI cases.

We found malnutrition to be an important risk factor for ARI-related fatality among both young children and adults. Several other studies have similarly reported on the link between malnutrition and respiratory deaths. Two systematic reviews from studies in low- and middle-income countries found that both severe malnutrition (OR range: 2.5–15) and moderate malnutrition (OR range: 1.2–36.5) significantly increased the risk of mortality among children with pneumonia and ARI [27]. However, data on the role of nutrition and ARI-related mortality in adults are more limited. In one Kenyan study, BMI was not a significant risk factor for mortality among adults with acute pneumonia [16]. However, low BMI may be a marker for serious underlying illness. One study among Navajo adults found that low BMI was associated with the risk of invasive pneumococcal disease [28]. In selected studies, obesity was also found to be a significant risk factor for death during the 2009 H1N1 influenza pandemic [8, 10].

Further research is needed to better understand how nutritional status, particularly malnutrition, affects risk of mortality among adults with ARI. Malnutrition could be associated with a poor immune response, making an individual more susceptible to infections including pneumonia, and infections can also contribute to malnutrition [29]. In Guatemala, the national nutritional survey *Nacional de Salud Materna Infantil (ENSMI)* published in 2017 reported that 46.5% of children < 5 years of age experience chronic malnutrition [30]. Importantly, malnutrition is a modifiable risk factor and improvements in nutritional status through interventions such as programs targeting improved diets and vitamin A and zinc supplementation can reduce the risk of developing pneumonia, as well as the risk of death among children with pneumonia. One study estimated that an intervention that prevented 40% of childhood cases of malnutrition in developing countries could lead to a 5.1–13.3% reduction in deaths from pneumonia, depending on the region [31].

Breastfeeding was protective against death in patients aged < 2 years in this study, even after adjusting for malnutrition, providing evidence of the benefits of breastfeeding beyond improved nutrition. Similarly, a multicentre cohort study in low- and middle-income countries found that infants aged 6–26 weeks not breastfed had an adjusted hazard ratio of 32.7 (95% CI: 6.8–157.2) for ARI-specific mortality compared to those exclusively breastfed, although this was a community-based study in contrast to our hospital-based study [32]. A systematic review including community- and hospital-based studies from 39 low- and middle-income countries found that inadequate breastfeeding practices were significantly associated with an increased risk of ARI-related death (OR: 1.8, 95% CI: 1.2–2.0) [6]. Maternal antibodies against infectious causes of respiratory disease can help reduce the severity of illness in the breastfeeding child [33]. Promotion of exclusive breastfeeding is an important public health intervention for reducing the burden of respiratory morbidity and mortality among young children. High utilization of outpatient care among pneumonia and influenza-like illness patients have been reported in Guatemala so community-based providers should be empowered to provide appropriate public health education and interventions such as the promotion of nutrition, breastfeeding and vaccination for the control of ARI [34].

We also found poverty to be associated with fatality among young children, which may reflect a wide range of inter-related factors including baseline health status, environmental exposures, and access to medical care. A systematic review found that low socioeconomic status was associated with an increased risk of ARI-related death among children aged less than 5 years (OR: 1.6, 95% CI: 1.3–2.0) [6]. Although poverty is not an easily modifiable risk factor, poverty reduction efforts could reduce the burden of ARI-related deaths as well as deaths from other diseases closely linked to socioeconomic status.

Comorbidities have been shown to be risk factors for ARI death among both children and adults. Among children, the most commonly reported comorbidity linked to ARI death is heart disease [6, 35–37], as was found in our study among children < 2 years. Among adults, most studies assessing risk factors for ARI-related death have focused on patients hospitalized with influenza. These studies have identified a range of comorbidities associated with fatality, although diabetes is commonly reported to have the strongest association [7–12]. Diabetes was not a risk factor for death in our study. We found kidney disease to be the only comorbidity associated with fatality, similar to another finding reported in a study on influenza-related fatalities [11].

Endpoint pneumonia was more common among patients with fatal ARI cases in both children aged < 2 years and adults. Endpoint pneumonia is considered a proxy for bacterial pneumonia in children [20] and has been used as an outcome for clinical trials and observational studies of household air pollution [38] and of vaccines against *Haemophilus influenzae* type b and *S. pneumoniae* [39]. Although experience with standardized CXR interpretation in adults is more limited, data from this same surveillance platform in Guatemala suggest that it can be used to identify adults more likely to have pneumococcal pneumonia [40]. We did not find *S. pneumoniae* isolated by blood culture or detected through urine antigen testing to be associated with death; however the number of positive results were limited, and blood culture is insensitive for detecting *S. pneumoniae* [41]. Nonetheless, the importance of endpoint pneumonia suggests that *S. pneumoniae* may be an important cause of ARI-related death in Guatemala. The 13-valent pneumococcal conjugate vaccine was introduced in Guatemala in 2011. It has been found to be protective against endpoint pneumonia, so although coverage remained fairly low during the study period, increased uptake of the vaccine could lead to reductions in pneumonia burden and potentially ARI deaths [42].

RSV is a leading cause of respiratory illness and death among children globally [43, 44], and was the most commonly detected virus among both fatal and nonfatal ARI cases in children < 2 years old in this study. Despite the well-recognized burden of RSV, we found it to be significantly more common among ARI patients discharged alive (43%) than among patients with fatal ARI cases (30%). This finding may be a reflection of the comparison group used for analysis and the insensitivity of available tests for bacterial causes of respiratory illness in children [45]. For example, if *S. pneumoniae* were relatively more common among RSV-negative patients than RSV-positive patients, and *S. pneumoniae* were more strongly associated with death than RSV, then RSV detection may appear to be protective against death; however the insensitivity of tests for *S. pneumoniae* limits our ability to test this hypothesis. Many ARI-related deaths among children occur at home without seeking medical care [46]. If children with RSV infection were less likely to receive care at the hospital (for example, due to absence of fever among many RSV case-patients) compared to children with other respiratory etiologies, then RSV-related deaths might be under-represented in our study. Overall ARI pathogen etiology results are discussed in more detail in Verani et al. and found to be similar to more recent respiratory disease surveillance studies [17, 47].

This study has limitations that should be acknowledged. Data on at-home deaths after hospital discharge were not systematically collected among all patients and this could have resulted in misclassification; some discharged alive may have died at home as a result of their illness and some discharged in a moribund state may have survived. Some characteristics that have been identified as risk factors for death among pneumonia patients in other studies were not adequately measured in this study to assess their possible role, including factors such as low birthweight, other infectious diseases such as malaria or measles, environmental factors, and poor water, sanitation and hygiene access/practices. Pathogen detection was limited by lack of specimens from the site of infection (e.g. inside the lung), available diagnostic tools, lack of blood culture sensitivity, and inability to use urine antigen testing in children because nasopharyngeal colonization is common and may lead to false positive results [19]. Improved rapid point of care diagnostic tests for viral and bacterial respiratory tract infections are needed to better understand the role of each pathogen and improve management outcomes [48]. It is also worth noting that the ARI case definition could have affected results although it has been found in other studies to have high sensitivity and capture more than 67% of RSV-associated hospitalizations [49]. Other variables, including selected vaccinations, end-point pneumonia, and urine antigen testing had significant missing data, which could have biased results. Lastly, our comparison group was patients hospitalized with ARI, rather than healthy controls, so we were not describing factors associated with the development of ARI or population-based incidence of ARI mortality, but rather those associated with death among ARI patients.

## Conclusions

Efforts to control respiratory disease and end preventable deaths should be based on evidence, particularly in low- and middle-income countries where data are often more limited despite a higher burden of morbidity and mortality. Our results identify important risk factors for ARI-related death and highlight the importance of strategies to promote improved nutrition and breastfeeding, as well as the prevention and management of chronic illnesses such as kidney and heart disease. Increasing coverage with the pneumococcal conjugate vaccine is expected to lead to reductions in ARI-related mortality in this population. Our findings underscore the need to use a wider approach for prevention and control of ARI fatality, including consideration of the factors we highlight above and as reflected in and the United Nations Sustainable Development Goals [50].

## Abbreviations

ARI: Acute respiratory infection
BMI: Body mass index
CI: Confidence interval
CXR: Chest radiograph
ICU: Intensive care unit
NP: Nasopharyngeal
OP: Oropharyngeal
RSV: Respiratory syncytial virus
UVG: Universidad del Valle de Guatemala
VICo: Vigilancia Integrada Comunitaria
WHO: World Health Organization
